# Green pesticide: Tapping to the promising roles of plant secreted small RNAs and responses towards extracellular DNA

**DOI:** 10.1016/j.ncrna.2021.02.001

**Published:** 2021-02-13

**Authors:** Karlia Meitha, Rizkita Rachmi Esyanti, Iriawati, Ristag Hamida Hanisia, Rohyani

**Affiliations:** School of Life Sciences and Technology, Institut Teknologi Bandung, Jl. Ganesha No. 10, Bandung, 40132, West Java, Indonesia

**Keywords:** Extracellular genetic materials, Elicitor, Plant response, Signalling, Plant immune, exDNA, Extracellular DNA, exRNA, Extracellular RNA

## Abstract

The diverse roles of non-coding RNA and DNA in cross-species communication is yet to be revealed. Once thought to only involve intra-specifically in regulating gene expression, the evidence that these genetic materials can also modulate gene expression between species that belong to different kingdoms is accumulating. Plants send small RNAs to the pathogen or parasite when they are being attacked, targeting essential mRNAs for infection or parasitism of the hosts. However, the same survival mechanism is also deployed by the pathogen or parasite to destabilize plant immune responses. In plants, it is suggested that exposure to extracellular self-DNA impedes growth, while to extracellular non-self-DNA induces the modulation of reactive oxygen species, expression of resistance related genes, epigenetic mechanism, or suppression of disease severity. Exploring the potential of secreted RNA and extracellular DNA as a green pesticide could be a promising alternative if we are to provide food for the future global population without further damaging the environment. Hence, some studies on plant secreted RNA and responses towards extracellular DNA are discussed in this review. The precise mode of action of entry and the following cascade of signaling once the plant cell is exposed to secreted RNA or extracellular DNA could be an interesting topic for future research.

## 1 Introduction

The world population is growing rapidly that continuously increases food demand. For instance, a survey by Indonesian Bureau of Statistics (BPS) in 2016 showed the demand of staple foods, such as corn, increased by 20.95% from 2015 to 2017 [1]. In addition, the increase in consumption was also observed in the horticultural food sector such as chili, banana, sweet potato, etc. However, fulfilling the global food demand is still problematic due to compromised productivity that, among others, is caused by weeds, pests and pathogens.

Aggressive weeds are one of the limiting factors in crop production since they absorb nutrients and water faster than the main crops [2]. Pitoyo [3] states that weeds can decrease rice productivity by 6up to 87%. Moreover, the cost of weed controls in rice could be as high as 50% of the total production cost [4]. Severe pest and pathogen infestation can result in loss of production, a decrease in quality, which leads to a decrease in farmers’ income. For instance, rice-ear bug (*Leptocorisa oratoria*) is a harmful pest, causing up to 50% production loss in a certain condition [5]. Pathogen attacks in crops may be caused by various microorganisms such as virus, bacteria [6], and fungi [7]. Fusarium are notorious fungi that are responsible for most fungi-related damages in crops, such as *F. oxysporum* in banana; *F. solani* in chili, vanilla, cocoa, and other crops [7]. *F. oxysporum* is also known to be the cause of major economic losses in the production process of horticultural crops [8].

In order to avoid the loss caused by weeds, pests, and pathogens, there are three alternative methods to minimalize the losses: physical, chemical, and/or biological control. Till date, the most common methods used to control weeds are physical and chemical, while to control pests the methods are mainly chemical. The manual weed control method is laborious and costly. Meanwhile, the chemical method using herbicide and pesticide is more efficient, faster, and cheaper, but comes with great environmental costs. Herbicide use can result in decade-long toxicity due to its difficulty to degrade and, at the same time, its detrimental capacity to reduce the soil's organic materials, water retention capacity, soil conservation, and fertility. Herbicide can also reduce related biodiversity, e.g., plants, fish, and birds, while also affecting the soil microorganisms' composition. Acute toxicity due to long exposure can lead to health problems, from skin rashes to death [9]. Direct and non-direct exposure of pesticides on food and water can induce health risks. Moreover, pesticides also pose negative effects to the environment (e.g., contamination of water, soil, and air; loss of wildlife, fish, plants, and other non-target organisms) [10].

Hence, the biological method is the safest to control pest, pathogen, weed, parasite, nematode, and diseases. Some widely practiced biological methods are planting disease-resistant cultivars, adding probiotic organisms in the growing medium, and others. However, some of these defensive methods in controlling weeds, pests, and pathogens, especially physical and chemical methods, are known to be less specific and can also affect the non-target organisms. Those methods work by either killing targeted organisms or rendering plant defense a faster activation time than the non-treated plants.

Recent studies found that the transfer of small-RNAs is involved in plant-pathogen interaction. Horizontal transfer between host and parasitic plants was documented in *Cuscuta* parasitism [[11], [12], [13]], while vertical transfer was documented in the interaction of plant and pathogenic fungi [14,15]. Plants naturally secrete interspecific small-RNAs as a part of their defense system to silence pathogen mRNAs. Conversely, pathogens also send their small-RNAs to repress host genes related to immunity and defense. It is suggested that mRNAs secretion from plants is transferred in extracellular vesicles to avoid degradation from abiotic condition or digestion by extracellular RNAse [16].

The application of extracellular non-self-RNA (non-self exRNA) to plants led to the induction of the upsurge of reactive oxygen species (ROS), activation of Mitogen-Activated Protein Kinase (MAPK), expression of defense genes, and callose deposition [17]. Similarly, Niehl et al. [18] suggest that a 746 bp synthetic dsRNA analogous to self DNA could induce plant-triggered immunity responses such as the activation of MPK6 and MPK3, ethylene production, and also inhibited germination in Arabidopsis. Bacterial RNA possesses distinct secondary structures that could be one of the keys in dictating response from specific plants. Further, Seybold et al. [19] found that RNA from a pathogenic bacterium, *Pseudomonas syringae* pv. tomato DC3000, could improve innate immune response and reduce the level of infection in tomato plant.

Interestingly, coordinated responses were observed following an exposure to extracellular self-DNA (self exDNA). A study conducted by Mazzoleni et al. [20,21] showed that a certain concentration of the self exDNA could impede the growth of organisms from various taxa. The exposure effect of self exDNA was species-specific; it only affected the same species as the DNA source. Aside from having a self-impacting property, the application of DNA from other organisms (i.e., pathogen) could respectively increase the defense related mechanisms in plants [22]. However, plant recognition of the DNA and RNA from extracellular environment is still vague [18].

Thus, a proper source and dose of RNA or DNA treatment to plants could result in: (1) growth inhibition; or (2) specific induction of defense system by acting as an elicitor for plants and by repressing expression of detrimental genes in pests or pathogens. In this review, we discuss natural interactions between plants and extracellular RNA/DNA that are present in the extracellular environment or deliberately sent out of the cell when interacting with other organisms. Furthermore, this review highlights the potential use of RNA/DNA-based pesticides on alleviating disturbances in the farming process based on the interaction of the RNA/DNA and plants.

## 2 Interspecific secreted RNA

Once thought to only play a role in the process of endogenous molecular signaling, plant small-RNAs are now gaining popularity as an agent in cross-kingdom communication [[23], [24], [25], [26]]. Recent studies show that signaling between pathogens and their host plants involves the transfer of small RNAs. Horizontal transfer is documented between the parasite plant, *Cuscuta* sp., and their hosts [11,25,[27], [28], [29], [30]], whereas vertical transfer is documented in fungi and plant interaction [[14], [15], [16],^31^]. Small-RNAs, usually in the range of 21–24 nucleotides, in eukaryotic organisms are short non-coding regulatory elements that induce RNA interference (RNAi) mechanisms. Axtel [32] classified small-RNAs in plants based on their biogenesis and function; the first class are those generated from single stranded precursors with a hairpin structure (hpRNA) and the second are from double stranded RNA (dsRNA) precursors. In plants, two major types that are transported out of the producing organism to interacting species are microRNA (miRNA), a member of the first class, and a secondary small interfering RNA (siRNA) that belongs to the second class. Endogenously, miRNAs target transcripts distinct from their own precursors, while siRNAs target those from the same loci where they originated [33,34]. The small-RNAs interspecific or even interkingdom targeting mechanisms are currently an interesting field of research.

miRNA is thought to have an important role in plant development, stress tolerance, and disease resistance [35]. Several reports suggest the role of this circulating miRNA as a new mode of communication between different types of cells/tissues, in which the miRNA secreted from one tissue exerts a regulatory effect on mRNA targets in different tissues [35]. It is also known that miRNA derived from plants can pass through the digestive tract and enter the blood circulation [36].

The transfer of small-RNAs between plants and their pests are bidirectional as systematically reviewed in Refs. [26,37,38]. Plants naturally secrete interspecific small-RNAs as part of their defense system to silence pathogens mRNAs, known as host-induced genes silencing (HIGS). Conversely, pathogens also send small-RNAs to repress host gene expressions related to plant immunity. The plant mRNAs are cargo of extracellular vesicles [16], a strategy to ensure safety avoiding degradation due to abiotic conditions or the presence of extracellular RNAse. Likewise, extracellular delivery of small-RNAs from bacteria, fungi, and protists are proposed to involve vesicles [39]. Understanding the mode of action and machineries involved in communication triggered by small-RNAs between plants and their pathogens is crucial to develop novel strategies in crop protection. Hence, noteworthy studies on exchange of small-RNAs between plants and pathogens (Table A.1) will be discussed here.

**Table A.1:** Interspecific RNA communication between plants and their parasite or pathogen

Interspecific RNA signaling	Host	Parasite/pathogen	Reference
Bidirectional exchange of transcripts	*A. thaliana*	*C. pentagona*	[11]
	Tomato	*C. pentagona*	
Transcripts transfer from host to pathogen	*A. thaliana*	*C. reflexa*	[40]
Host mRNA transfer to parasite	*A. thaliana*	*C. pentagona*	[29]
	Tomato	*C. pentagona*	[30]
Host mRNA transfer to parasite	*Pumpkin*	*C. pentagona*	[30]
Host siRNA transfer to parasite	Tobacco	*C. pentagona*	[27]
Parasite miRNA transfer to host	*A. thaliana*	*C. campestris*	[25]
	Tobacco		
Host small-RNAs transfer to pathogen	*A. thaliana*	*B. cinerea*	[26]
Host hpRNA transfer to pathogen	Potato	*P. infestans*	[31]
Pathogen small-RNAs transfer to pathogen	*A. thaliana*	*B. cinerea*	[41]
Host small-RNAs transfer to pathogen	*A. thaliana*	*P. capsici*	[14]

### 2.1 Small-RNA biogenesis

Biogenesis of the first class small-RNAs involve RNA polymerase II to assist the synthesis of self-complimentary RNA, a precursor of hpRNA. Once the hpRNA is formed, it will be digested by Dicer-like endonuclease (DCL) family resulting in shorter single stranded RNAs, termed miRNAs, whereas the production of second class small-RNAs involves dsRNA and multiple DCLs, resulting in the production of small interference RNAs (siRNAs, a collective term). Then, miRNAs or siRNAs will form a complex with Argonaute 1 (AGO1), a family of endonuclease that cuts target RNAs. The complex will further undergo one of these pathways: (i) binds to complement mRNA that causes degradation and translational repression, (ii) binds to complement long non-coding RNA (lncRNA) that attracts other classes of endonuclease and expression of lncRNA target retained, (iii) forms lncRNA – mRNA complex to promote sequestration and turnover [38]. Digestion results from pathway (i) and (ii) could also bind to AGO1, forming miRNA-AGO1 or siRNA-AGO1, and interact with RNA target. This complex will then stimulate RNA-dependent RNA polymerase 6 (RDR6) to generate dsRNA. DCL2 will recognize the dsRNA and cut it into fragments of 21 nucleotides, or DCL4 will cut it into fragments of 22 nucleotides. These short fragments of RNA are termed as secondary siRNAs, which will then go through pathway (i), (ii), or (iii).

#### 2.1.1 Plant to plant: Horizontal transfer

Horizontal transfer of mRNAs and small-RNAs between plant species is recorded in the interaction between *Cuscuta* species, parasitic plants, and hosts. *Cuscuta* genus consists of about 200 species, specializing in living parasitically by connecting to their hosts plant via vascular system to extract water, nutrients, and metabolites [25]. *Cuscuta* form haustorial connections, in which their haustoria intrusively invade the phloem and xylem of their hosts. The transfer of macromolecules in this parasitic symbiosis is predicted to involve plasmodesmatal and phloem connections [28].

Parasitism of *Arabidopsis thaliana* [11,29], pumpkin [30] and tomato [11,30] by *Cuscuta pentagona* involves horizontal mRNAs transfer. Transcriptome composition in the interface area of *C. pentagona* attachment to their respective host shows 51% similarity to *A. thaliana* and 86% to tomato. Further, 0.6% of transcripts in *A. thaliana* and 0.38% in tomato stem, adjacent to the attachment area of *C. pentagona*, are unique to the parasitic plant [11]. Roney and colleagues [30] identified three mobile mRNAs belonging to pumpkin (*Cucurbita maxima* Duch.) and ten belonging to tomato (*Solanum lycopersicum*) in *C. pentagona* when parasitizes the respective plant, as assayed by reverse transcriptase PCR. However, although GIBBERILLIC ACID-INSENSITIVE (GAI) transcripts were detected in both pumpkin and tomato, it was only transferred to *C. pentagona* grown on tomato [30,42]. *C. reflexa* parasitism to *A. thaliana* also involves a transfer of over 2,000 distinct transcripts from the host [40], a lower number compared to over 9,000 in *C. pentagona* parasitism [11].

In order to repress *C. pentagona* parasitism to plant, Alakonya [27] tested the consequence of transforming tobacco to produce siRNA targeting two genes in *C. pentagona*. SHOOT MERISTEMLESS-like (STM) is a member of KNOTTED1-like HOMEOBOX1 (KNOX1) gene family that regulates the indeterminate identity in the shoot apical meristem in angiosperms [43] and also during the formation of haustoria in *C. pentagona* [27]. Interestingly, siRNA production controlled by a vascular promoter (SUCROSE-PROTON SYMPORTER2) in tobacco to target interspecific STM gene in *C. pentagona* was able to reduce the vigor of the parasite grown on the transformed plant. The study also confirmed that siRNA targeted STM was undetected in *C. pentagona* grown on wild type tobacco, suggesting that growth inhibition was due to siRNA mobility from host to parasite.

Conversely, transfer of miRNA from *Cuscuta campestris* to hosts was demonstrated in the interaction with *A. thaliana* and tobacco [25]. Foreign miRNAs, with 22 nucleotides in length, were found abundantly in the area of *C. campestris* attachment to the aforementioned hosts. *C. campestris* miRNAs target *A. thaliana* mRNAs, which further lead to cleavage of mRNAs, decreased mRNAs accumulation, and generation of secondary siRNA in host. However, this was not found when the two loci that encode target mRNAs in *A. thaliana* were mutated, indicating specificity of *C. campestris* miRNAs to host the gene. Thus, the evidence supports that interspecific RNA transfer between plants is a process that is bidirectional and unique/specific to each parasite-host interaction. In the future, application of RNA-based pesticide to control *Cuscuta* species could be formulated as it is specific and natural.

#### 2.1.2 Plant-fungi: Vertical transfer

One of plant strategies to repress virulence genes when combating fungal pathogenicity is by excreting sRNAs. A well-documented phenomenon in plant pathogenesis by *Botryris cinerea* [26,44], *Phytophthora infestans* [31] and *Phytophthora capsici* [14]. Cai and colleagues [44] identified that mutating the machineries for sRNAs generation in *A. thaliana*, such as the endogenous nuclease dcl2/3/4 and RNA polymerase rdr6, caused an increased susceptibility towards *B. cinerea*. This could due to the inability of *A. thaliana* to produce 42 interspecific small-RNAs that was detected in *B. cinerea* when infecting the wild type plant. Interestingly, 31 out of 42 of these small-RNAs are protected during delivery in exosomes, from extracellular nuclease as well. However, the detailed mechanisms of membrane vesicles and cell membrane recognition and entry of interspecific small-RNAs to plant or pathogen cells are not yet fully understood. *Botritys cinerea* genome encodes two DCL-like genes, *DCL1* and *DCL2*, and mutation to both genes lead to decreased pathogenicity and smaller lesion in the infected plants. Thus, generation of RNAi targeting fungal *DCL1* and *DCL2* in mutant *A. thaliana* and tomato increased resistance to *B. cinerea* [26]. Similarly, transformed potato generating a hpRNA targeting G protein β-subunit (*PiGPB1*) gene in *P. infestans* showed an increased vigor under the infection of the fungi [31], a promising alternative for green fungicide.

On the contrary, fungal pathogens also send small-RNAs to their hosts, a bidirectional interaction to weaken each other's guard. *Botrytis cinerea* delivers small-RNA as an effector to silence host genes, identified as siR37, when infecting *A. thaliana* [41]. There are 15 genes with repressed expression during *A. thaliana* infection by the fungi, and two of them were significantly reduced when siR37 was applied transiently to the leaves. Further, A. thaliana mutants, each with a defect in siR37 target genes, *FEI2, PMR6,* and *WRKY7,* displayed attenuated defense to *B. cinerea* infection. The *FEI2, PMR6,* and *WRKY7* genes in *A. thaliana* encode a leucine-rich repeat receptor kinase, a pectin lyase, and an immune-related transcription factor, respectively.

A reciprocal tactic in *Phytophthora capsici* pathogenicity to *A. thaliana* involves interkingdom transfer of small-RNAs and effector protein. *A. thaliana* has an established role in delivering small-RNAs to increase defense towards *P. capsici* such as to repress *Phyca-_554980* gene, encodes U2-associated splicing factor and constitutively expressed. This was achieved by the generation of siRNA-1310, 21 nucleotides long, that repress *P. capsici* infection. However, *P. capsici* is also able to disrupt this strategy by transferring an effector, Phytophtora suppressors of RNA silencing (PSR), reducing small-RNA generation in the plant host which lead to plant disease [14].

## 3 Extracellular DNA

Extracellular DNA (exDNA) is released by dead cells, viral DNA, or fragmented DNA that is secreted by metabolically active cells [45]. Following cell death, DNA molecule could undergo several processes such as natural transformation, degradation, preservation, and decomposition. Both prokaryotic and eukaryotic cells are capable of transporting exDNA. Genetic transformation can occur in microbes by drawing in free DNA from the environment [46]. Eukaryotic organisms such as plants can also affiliate organic molecules, including proteins and DNA into the roots [22]. The role of exDNA is also known as one of the defense components of the root extracellular traps [22,47]. A fluorescence-labeled 25 bp of DNA was detected inside the root cells (including the root hair) and pollen tube [48]. DNAse treatment in *Pisum sativum* roots can reduce resistance to fungi pathogen *Nectria haematococca*, indicating that exDNA may play a role in plant defense to pathogen [22].

Ferrusquía-Jiménez et al. [49] proposed the relevant role of extracellular DNA as a plant damage-associated molecular pattern (DAMP) for improving crop resistance towards pests. The mechanism involves: (i) inhibitory activity as the biologic control of the pests and/or (ii) elicitation activity to induce immune responses such as plant vaccines. Equally, Quintana-Rodriguez et al. [50] suggested the future of exDNA as plant vaccines along with other potential DAMP such as cell wall fragments, sucrose, proteins, peptides and volatiles. Based on the source of species, exDNA is categorized into self exDNA and non-self exDNA. The recognition and extraction of free DNA (i.e., self or non-self exDNA) from the environment, can generate distinct impacts on the cell activity. However, the self-recognition mechanism and the extracellular signaling process of exDNA are still unknown.

### 3.1 Extracellular self-DNA

Mazzoleni et al. [20] observed the inhibition effect of extracellular self-DNA (self exDNA) when growing plants in decomposed leaf-litter of the same species. Further examination revealed that the autotoxicity was one of the consequences of plant contact with self exDNA fragment in a certain concentration. To test the specificity of response, the exDNA treatment was applied to organisms from various taxa. The study was conducted by applying self and non-self exDNA into microscopic organism colony, the seed of *Acanthus mollis* and fly larvae of *Sarcophaga carnaria*. The result showed that the DNA from heterologous organisms did not affect growth. However, the application of DNA from the same species in a particular concentration can negatively impact the organism growth.

Mazzolenni's studies [20,21] demonstrated that self exDNA, in a certain concentration, could inhibit the growth of organisms from various kingdoms. The effect of self exDNA exposure is species-specific in that it only affects species that share similar DNA and does not significantly impact other unrelated species. Duran and Heil [51] also revealed the self-inhibitory mechanism in plants. These studies suggest that such mechanism may prevent intra-specific competition and possibly act as an intraspecific stress signal. To put it simply, a high concentration of self exDNA provides information on occurring damage and death in other individuals of the same species. This signal is then relayed to the surrounding plant or seed from the same species to inhibit their growth [52].

Although the self exDNA exposure effect was obvious on plant growth, the mechanism at the molecular level, including the recognition of self exDNA, has not been clearly described. Bhat and Ryu [53] proposed four hypotheses of possible exDNA and exRNA perception or recognition in plants. The first hypothesis suggested the presence of a membrane-bound exDNA/exRNA receptor which is able to recognize a specific microbial DNA/RNA and triggers cascading-signals through the post-translation modification. The second mentioned the exDNA/exRNA transporter channel bound to a membrane which binds and transports exDNA/exRNA fragments to the cytoplasm. Third, exDNA/exRNA internalization is made through vesicles recognized by cytoplasmic sensors or directed to interference with the RNA. The fourth hypothesis suggested the intracellular exDNA/exRNA censors mediate a surveillance function to detect foreign nucleic acids. Further studies to test each hypothesis are needed. Nevertheless, it is understood that exDNA and exRNA trigger plant signaling and response.

Homologous exDNA recognition can affect cell functions at different levels, i.e., signaling, gene expression mechanism, and response formation (e.g., plant growth inhibition). In their study on self exDNA of lima bean (*Phaseolus lunatus*) and maize (*Zea mays*), Barbero et al. [6] showed that self exDNA significantly induced the membrane depolarization and Ca^2+^ flux increase, whereas this was not shown in non-self exDNA. This relates to the plant and biotroph interaction in the sense that the plasma membrane is the first layer of recognizing external molecules, which will lead to the alteration of the potential membrane (Vm), also known as electrochemical gradients between the inner and outer part of the cell [54]. This induces ionic imbalance and modulations of the channel in the plasma membrane, resulting in Vm alteration that involves variations in cytosolic Ca^2+^ concentration. In this interaction, Vm variations are dependent on Ca^2+^ openings of inward K^+^ channels, which will reduce the Vm to a depolarization state [55]. This initiates gene expressions and plant response [56,57]. Changes in the potential membrane and Ca^2+^ flux confirm that self exDNA induces early signaling that leads to damage response in plants.

Furthermore, Duran-Flores and Heil [51] also showed that self exDNA exposure on *Phaseolus vulgaris* L.’s leaf induced the formation of ROS and resistance-related responses. This did not occur when the plant was exposed to non-self exDNA. Both the plant and *P. vulgaris* suspension cultured cells exposed to self exDNA showed an increase in H_2_O_2_ generation and MAPK activation, followed by a decrease in bacterial (i.e., *Pseudomonas syringae*) infection and an increase in indirect defense against herbivores (i.e., extrafloral nectar secretion).

ROS can play a role in cascade signaling process and assist in defense against pathogens as long as their concentrations are within tolerance [58,59]. A study on self exDNA by Vega-Munoz et al. [60] suggested an alternative explanation on the changes of DNA methylation (epigenetics) as a result of self exDNA and non-self exDNA exposure. Methylation patterns of DNA always change due to abiotic, pathogenic, and infection-induced stresses, or after treatment with salicylic acid. Changes in DNA methylation was reported following the use of salicylic acid in *Pennisetum glaucum*, promoting defense pathways [61]. This could be due to the difference on methylated genome area, which affected differential gene expressions that were linked to the oxidative bursts and the production of secondary metabolites related to coping with stress conditions.

Study conducted by Vega-Muñoz et al. [60], show that DNA exposure from the same clade significantly results in the hypomethylation of CpG DNA. Changes in DNA methylation levels could be seen as an epigenetic mechanism to control gene expressions. A higher concentration of self exDNA (200 mg/mL) did not proportionally result in a change of CpG DNA methylation levels, although this concentration demonstrated a significant change in the inhibitions of seed germination, root growth, and expression of pal, superoxide dismutase and catalase genes, as well as the increase of phenylpropanoids production in lettuce seedlings [60]. These genes correlate with oxidative stress signaling and the production of secondary metabolites (phenylpropanoids) to cope with stress conditions [62]. The level of changes in DNA methylation also increased the production of secondary metabolites related to defense responses to stresses. The result showed that the effect of fragmented extracellular DNA depends on phylogenetic relations that could promote epigenetics and biochemical modulation in plants.

Responses in potential membrane change, Ca^2+^ flux, increased H_2_O_2_ and MAPK production, and DNA methylation indicate that self exDNA acts as the Damage-Associated Molecular Patterns (DAMP). The term DAMP initially refers to the hydrophobic part of a biological molecule that originates from a dead host cell and pathogen, which triggers immunity when exposed [63]. Currently, DAMP is used to refer to distress signals from a damaged host cell [64]. If tissue damage occurs, DAMP will promptly induce responses from pattern recognition receptors (PRRs) among others through Ca^2+^ concentration change, as well as ROS and MAPK increase [51].

During infection, damaged tissues urge cells to form small compartments and release intracellular molecules to the extracellular environment [65]. These molecules are recognizable by the surrounding (intact) cells as DAMPs which trigger self-destructing recognition, thus inducing immunity on the damaged organism. An endogenous molecule that is identified as DAMP provides an activation signal through the self-damaged recognition pathway, whereas exogenous molecules related to pathogens (PAMP) activate non-self-recognition pathway [66]. These would then lead to a cascade of responses involving the expression of the instant defense-related genes [19,[67], [68], [69], [70]].

Due to its ability to inhibit growth, self exDNA is proposed as an alternative for the natural herbicide to control specific crop weeds. Self exDNA significantly reduced growth only when applied intra specifically, minimizing the risks of repressing non-pathogenic organisms. In addition, exDNA is a natural component of the environment, which is biodegradable and pose minimum risks compared to chemical herbicides. This notwithstanding, further study is still needed to evaluate the phenomenon better and doing so leads to developing more appropriate technology.

### 3.2 Extracellular non-self DNA

In mammals, it is identified that among others, there are a few receptor proteins, such as TLR9, ZBP/DAI, and RAGE, which can receive signals from DNA/RNA and trigger an immune response through IFN-I-dependent signaling pathway [71]. TLR9 is predicted to play a role in distinguishing self and non-self exDNA, due to its ability in recognizing CpG motive in the sequence. In plants, self exDNA could be categorized as DAMP, while non-self exDNA is categorized as PAMP. Plants are known to have plenty PPR proteins that are able to detect a variety of DAMP/PAMP and trigger signaling through Ca^2+^ influx, ROS production, and MAPK activation [71]. However, there is less information about which plant's PPR has a similar role as TLR9 in mammals, and whether there is a receptor in a plant cell that has a self/non-self recognition ability [71]. This section will discuss the effect of non-self exDNA on plant signaling pathway (Table A.2).

**Table A.2:** Signaling and response mechanisms in plants triggered by the treatment of extracellular self or non-self-DNA.

Treated species	Treatment	Producing species	Signaling and response mechanisms	Reference
*Phaseolus vulgaris*	Leaf homogenate,	*P.lunatus* and *P. coccineus*	Induced immunity related responses	[72]
*Phaseolus lunatus* and *Zea mays*	Self and non-self exDNA	*Spodoptera littoralis*	Significantly increased the membrane depolarization and Ca^+">2+^ flux in self exDNA treatment	[6]
Various taxa (microbes, fungi, protozoa, plants, insects)	Self and non-self exDNA	Various taxa	Concentration-dependent manner on growth inhibitory effect in self exDNA treatment and not significantly in non-self exDNA	[20,21]
Common bean (*Phaseolus vulgaris*)	Self and non-self exDNA	*P. lunatus*	Self exDNA inhibited growth primary root, significant increase of H_2_O_2_, activation of MAPK, reduced infection by the bacterial pathogen significantly more than non-self exDNA	[51]
Lettuce (*Lactuca sativa* L.)	Self and non-self exDNA	*Capsicum chinense* and *Acaciella angustissim*	Exogenous fragmented DNA as DAMP inducing changes in CpG DNA methylation and defence-related responses	[60]
*A.thaliana* vs *E.coli*	Non-self exDNA	Non metilated *E. coli* DNA	Induced H_2_O_2_ formation, callose deposition and activated FRK1 promoter	[17]
Wheat plant	non-self exDNA	cytosine-phosphate-guanine oligodesoxynucleotide motifs (CpG ODN)	Induced salicylic acid- and jasmonic acid-dependent signaling pathways and reduced infection from pathogen *Zymoseptoria tritici*	[73]
*Capsicum annuum*	Non-self DNA	fragmented DNA mixture of *Phytophthora capsici* L., *F.oxysporum* S., and *Rhizoctonia solani* K.	Increased total phenolic compounds, total flavonoids, and gene expression associated to plant defense such as phenylalanine ammonium lyase and chalcone synthase	[74]

In facing the signal of foreign molecules originating from pathogens, plants have two defense systems, i.e., PAMP-triggered immunity (PTI) and effector-triggered immunity (ETI). Both systems involve jasmonic and salicylic acid synthesis pathways, as well as the production of ROS and other mechanisms that will result in plant cell responses [75]. PTI is a response to a signaling pathway that depends on transmembrane PRR protein ability to recognize PAMP or DAMP molecules. ETI, on the other hand, is a response to a signaling pathway that operates at the intracellular level and relies on the cell's immunity gene [45]. A study conducted by Niehl et al. [18] showed that signals in the form of the nucleic acid will involve PTI signaling pathway and the protein SERK 1 (Somatic Embryogenesis Receptor-Like Kinase 1) as a co-receptor.

The presence of non-self exDNA from other species such as microorganisms could be interpreted as a signal for plants (PAMP) to activate plant immune system [53]. A study on the exposure of non-self exDNA as an elicitor on plant's defense system was conducted by Yakushiji et al. [17] on *A. thaliana*. The treatment consisted of *E. coli* DNA that were digested using *Eco*RI, *Sma*I, *Hap*II, *Hha*I, *Alu*I, *Sau*3AI, *Hae*III, or *Bam*HI enzymes. The fragments were then treated with CpG methyltransferase and S-adenosylmethionine to study the effect of DNA methylation. Genome DNA from prokaryotic cells possesses distinct unmethylated CpG motives, which are suspected to be one of the factors that determine recognition by plant cells as self or non-self exDNA. In the eukaryotic genome, CpG motives are mostly in a methylated state, although there is still a debate surrounding this, lending to the fact that mitochondrial DNA (mtDNA) has a similar structure as prokaryotic genome [71]. DNA sequences with CpG motive also bears the potential to develop into vaccine in the medicinal context [71]. The study by Yakushiji et al. showed that an addition of unmethylated oligonucleotide was able to increase H_2_O_2_ production, callose deposition that plays a role as a temporary cell wall, as well as activated FRK1 promoter [17]. Furthermore, the addition of ssDNA with CpG motive was able to increase the immunity of wheat towards fungal pathogen *Zymoseptoria tritici* [73]. Foliar treatment of CpG ODN on wheat with a concentration of 9.5 x 10^5^ g/L significantly reduced wound area, in addition to the modulated genes expression of *PR5*, *PR8*, *POX*, and *LOX2* [73]. *PR5* gene is one of the biomarkers for system acquired resistance (SAR) and is involved in the signaling of salicylic acid, whereas *LOX2* gene has a role in the signaling of jasmonic acid [73]. The most recent study [74] showed that the application of a cocktail of non-self exDNA (at 60 and 100 μg mL^−1^) was able to suppress disease severity and death percentage of *Capsicum annuum* infected by the mixture of these pathogens of *Phytophthora capsici* L.*,Fusarium oxysporum* S., and *Rhizoctonia solani* K. It is interesting that only within 24 h post foliar spray of pathogens exDNA, the immune response in treated plants was already improved [74]. Hence, extracellular DNA is suggested to also act as DAMP or PAMP, as they are competent to induce physiological and molecular changes leading to enhanced plant immune system.

Aside from the use of extracellular non-self-DNA as an elicitor, the transfer and integration of non-extracellular DNA to plant, even surpassing taxa, is not unusual. It is frequently studied in the evolutionary process of unicellular prokaryotes. However, gene transfer between multicellular organisms are less common. In plants, studies of gene transfer process were mainly focused on gene transfer from the plasmid of *Agrobacterium tumefaciens*. The gene transfer process in plants is facilitated by intercellular contact, wound, symbiotic relationship, and vectors such as virus, bacteria, and fungi. Mower et al. [12] reported an occurrence of a gene transfer between a host plant and its parasite. For instance, three genes originating from the parasite plant *Cuscuta* were found in the mtDNA of Plantago, which are *atp1, atp6*, and *matR*. Studies have also shown that gene transfer process plays a role in the evolution of Plantago [12].

Based on the aforementioned explanation, it is concluded that non-self exDNA acts as a signal for plant cells to improve defense by activating immunity system. This indicates that non-self exDNA has the potential to be used as an elicitor, increasing plant resistance when infected by the corresponding pathogen. The mechanism of perception of non-self exDNA in plant cells, however, has not been fully understood, and therefore there is a need for further study on this particular phenomenon, in a way that the application of non-self exDNA in plants as elicitor can be performed accurately.

## 4 Conclusion

**Fig. A.1:**
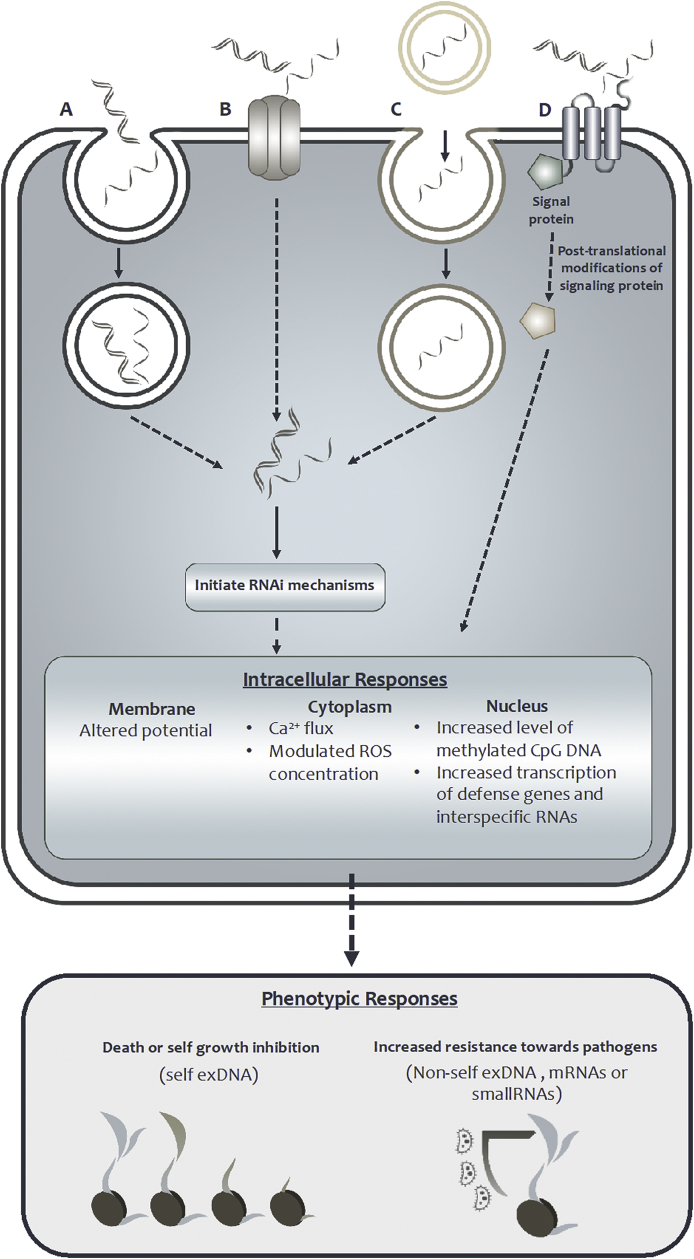
The proposed mechanisms of entry triggered cellular signaling cascade and phenotypic response of extracellular DNA or RNA exposure to plants. Prior to triggering intracellular signaling cascades; in (A), (B), and (C), the extracellular fragments of DNA or RNA enter the plant cell and disrupt RNA function in cytoplasm, while in (D), the fragments are recognized by a membrane-bound receptor. (A) and (C) involve internalization of the fragments in vesicles, but in (C), the fragments are also secreted in an extracellular vesicle. In (B), it is proposed that the DNA or RNA fragments could fit a designated channel to be transported into the cytoplasm. The triggered intracellular responses will then accumulate into phenotypic responses, such as growth inhibition, following self exDNA treatment or increased resistance to pathogens after treated with non-self exDNA, mRNA or small-RNAs.

The constantly increasing global food demand has brought negative consequences to the environment. Accumulation of toxic chemicals from pesticides lead to biodiversity loss in many parts of the world, which also reduced soil fertility limiting crop productivity in the following seasons. The application of DNA and RNA fragments is considered one of the promising solutions, due to their green nature and specificity in the mode of action. The proposed mechanisms of entry triggered signaling cascades and phenotypic responses of plants are summarized in Figure A.1. Exposure to extracellular self-DNA is documented to limit growth, while to non-self DNA has improved cellular processes related to pathogen resistance. Hence, it seems intriguing to propose the use of extracellular self-DNA as a green herbicide, repressing weeds by exposing to their own fragmented DNA. Also, it is considerable to use of short sequences of pathogens DNA as an elicitor to enhance plant immune system.

Transcripts exchange between plants and parasites or pathogens involving mRNAs and small-RNAs were recently uncovered. This mechanism is mostly known to repress gene expression in the interacting organisms that would benefit the producing species. For instance, during *Phytophthora capsici* infection on *A. thaliana*, the plant host delivers a 21 bp siRNA targeting a specific transcript in the pathogen [14]. Thus, it is worth to investigate the potential of developing this mechanism into a specific and green fungicide. Moreover, the use of delivery agent with anti-bacterial or anti-fungi properties could amplify the outcome, such as chitosan in suppressing *P. capsici* in *Capsicum annuum* [59]. In terms of cost, it is noted that the multinational agro-industrial companies charge as low as one USD per gram dsRNA [76]. However, this may not be the case for developing exDNA based pesticide, as also pointed out by Ferrusquía-Jiménez et al. [49], that the industrial production is still puzzling. On one hand, producing random fragments of DNA will require a massive preparation of the source species, reagents, and technical labor. On the other hand, producing through PCR based methods would need preliminary studies to confirm which sequence of fragments will trigger similar responses as the natural ones.

Furthermore, there are still unknown aspects of employing DNA or RNA as pesticide. Two of them are the detailed signaling mechanism and genetic changes that could be imposed. If we are to deliver the DNA or RNA into cellular compartment(s), then it is crucial to understand the mechanisms of uptake and intracellular transport, as well as how to improve fragment stability during application/delivery. Vogel [77] elaborated some delivery systems that have been tested in RNAi-based pesticides, involving nanoparticles, liposomes, carrier proteins, etc. Epigenetic changes following exposure to extracellular self-DNA was recorded in *Lactuca sativa* L., in the form of hypomethylation of CpG areas [60]. Furthermore, Dalakouras [76] also proposed three methods of DNA modifications that are mitotically stable, resulting from exogenous RNA application. In conclusion, the formulation of DNA or RNA-based pesticide requires a comprehensive consideration to fully benefit from both the biology of the plant and pathogen.
